# *Lactobacillus delbrueckii* subsp. *bulgaricus* 1.0207 Exopolysaccharides Attenuate Hydrogen Peroxide-Induced Oxidative Stress Damage in IPEC-J2 Cells through the Keap1/Nrf2 Pathway

**DOI:** 10.3390/antiox13091150

**Published:** 2024-09-23

**Authors:** Deyu Liu, Yingxue Yue, Lijun Ping, Cuicui Sun, Tingting Zheng, Yang Cheng, Guicheng Huo, Bailiang Li

**Affiliations:** 1 Food College, Northeast Agricultural University, Harbin 150030, China; 2 Key Laboratory of Dairy Science, Ministry of Education, Northeast Agricultural University, Harbin 150030, China

**Keywords:** *Lactobacillus bulgaricus*, exopolysaccharides, oxidative stress, IPEC-J2 cells, Keap1/Nrf2 pathway

## Abstract

*Lactobacillus delbrueckii* subsp. *bulgaricus* (*L. bulgaricus*) is one of the most commonly employed *Lactobacillus* in the food industry. Exopolysaccharides (EPS) of *Lactobacillus*, which are known to exhibit probiotic properties, are secondary metabolites produced during the growth of *Lactobacillus*. This study identified the structure of the EPS produced by *L. bulgaricus* 1.0207 and investigated the mitigation of *L. bulgaricus* 1.0207 EPS on H_2_O_2_-induced oxidative stress in IPEC-J2 cells. *L. bulgaricus* 1.0207 EPS consisted of glucose and galactose and possessed a molecular weight of 4.06 × 10^4^ Da. *L. bulgaricus* 1.0207 EPS exhibited notable scavenging capacity against DPPH, hydroxyl radicals, superoxide anions, and ABTS radicals. Additionally, *L. bulgaricus* 1.0207 EPS enhanced cell proliferation, reduced intracellular reactive oxygen species (ROS) accumulation, increased activity of superoxide dismutase (SOD), glutathione peroxidase (GSH-Px), catalase (CAT), and total antioxidant capacity (T-AOC) elevated the relative expression of CAT, SOD, HO-1, NQO1, ZO-1, and Occludin genes. Moreover, *L. bulgaricus* 1.0207 EPS improved the expression of Nrf2, *p*Nrf2, *p*Nrf2/Nrf2, and Bcl-2 proteins, while decreasing the expression of Keap1, Caspase3, and Bax proteins, with the best effect at a concentration of 100 μg/mL. *L. bulgaricus* 1.0207 EPS mitigated H_2_O_2_-induced oxidative stress injury in IPEC-J2 cells by activating the Keap1/Nrf2 pathway. Meanwhile, *L. bulgaricus* 1.0207 EPS exhibited the potential to decrease apoptosis and restore the integrity of the gut barrier. The findings establish a theoretical foundation for the development and application of *L.bulgaricus* 1.0207 and its EPS.

## 1. Introduction

Oxidative stress occurs when reactive oxygen species (ROS) levels surpass the cell’s capacity for scavenging in both intracellular and extracellular environments, leading to intracellular oxidative damage [1]. Excessive levels of ROS can induce oxidative stress, resulting in lipid peroxidation of cell membranes, oxidative damage to DNA and proteins, and alterations in the structure and function of intracellular molecules. These processes are implicated in the pathogenesis of various diseases [2]. Hydrogen peroxide (H_2_O_2_), a common ROS, is a pivotal mediator of oxidative stress-induced cellular injury in the intestine [3]. Probiotics, including various strains of *Lactobacillus*, have garnered significant attention for their potential health-promoting effects [4], particularly in mitigating oxidative stress [5]. Among these, *Lactobacillus bulgaricus* has been shown to exhibit antioxidative properties, partly attributed to its production of EPS [6].

*L. bulgaricus* is a prominent member of the lactic acid bacterium family, extensively utilized in the food industry, notably in the manufacture of yogurt and various other fermented dairy products [7]. *L. bulgaricus* is believed to offer health benefits by promoting the balance of intestinal flora [8], boosting immune system function [9], and potentially alleviating digestive issues [10]. EPS are secondary metabolites that arise during the growth and metabolic processes of *L. bulgaricus*, exhibiting a diverse array of structures [11]. EPS play a crucial role in modulating the immune system [12], scavenging free radicals [13], exhibiting antiviral properties [14], lowering cholesterol levels [15], and inhibiting the proliferation of cancer cells [16]. Moreover, EPS can alleviate oxidative stress through various mechanisms, including scavenging free radicals, enhancing antioxidant enzyme activity, bolstering cellular antioxidant capacity, and facilitating cellular repair processes [13,17].

The Keap1/Nrf2 pathway is a crucial cellular defense mechanism against oxidative stress and plays a central role in maintaining cellular redox homeostasis [18]. Under normal conditions, Keap1 binds to Nrf2 and facilitates its degradation via ubiquitination, thus keeping Nrf2 levels low in the cell. The Keap1/Nrf2 pathway is activated in response to oxidative stress, and Keap1 undergoes conformational changes, leading to the stabilization and nuclear translocation of Nrf2. Subsequently, Nrf2 binds to antioxidant response elements (AREs) in the promoter regions of its target genes, promoting their transcription and expression [19,20]. Several studies have indicated that EPS can activate the Keap1/Nrf2 pathway, thereby increasing the expression of antioxidant genes, and enhancing antioxidant enzyme activity. Specifically, *L. helveticus* KLDS1.8701 EPS mitigated hepatic oxidative stress by modulating the gut flora of mice through the regulation of the Nrf2/ARE signaling pathway. *L. plantarum* YW11 EPS alleviated oxidative stress in senescent mice by elevating antioxidant enzyme levels and mitigating D-galactose-induced oxidative stress.

This study analyzed the structure of *L. bulgaricus* 1.0207 EPS, and the mechanism by which EPS alleviates H_2_O_2_-induced oxidative stress in IPEC-J2 cells was investigated. Our findings may provide valuable insights into the development of novel dietary interventions or functional foods targeting gut health and oxidative stress-related gastrointestinal disorders.

## 2. Materials and Methods

### 2.1. Bacterial Strains and Cells

*L. bulgaricus* 1.0207 was sourced from the Key Laboratory of Dairy Science (KLDS) at Northeast Agricultural University (NEAU) in Harbin, China. Porcine intestinal epithelial IPEC-J2 cells were purchased from BeiNaChuangLian Biotechnology Research Institute (Beijing, China).

### 2.2. Extraction of EPS from L. bulgaricus 1.0207

EPS was extracted from *L. bulgaricus* 1.0207 following the method described by Mutamed et al. [21]. *L. bulgaricus* 1.0207 was inoculated into a sterile skimmed milk medium and fermented at 37 °C for 8 h. It was then centrifuged for 6 min at 4 °C at 8000 rpm and vacuum filtration. Afterwards, 80% (*w*/*v*) trichloroacetic acid (TCA) was added to the supernatant to a final concentration of 4% (*w*/*v*) and settled for 12 h at 4 °C, then centrifuged at 8000 rpm for 20 min. Three times the volume of anhydrous ethanol was added to the supernatant after rotary evaporation and settled for 12 h at 4 °C. Then it was centrifuged at 4 °C for 6 min at 8000 rpm, and the precipitate was collected. The precipitate was dissolved in deionized water and dialyzed for 48 h. Finally, the dialyzed solution was lyophilized to obtain the crude EPS.

### 2.3. Monosaccharide Composition Analysis

The monosaccharide composition of the *L. bulgaricus* 1.0207 EPS was measured by High-Performance Liquid Chromatography (HPLC) following the method of Qiao et al. [22]. NaOH (0.6 mol/L, 250 μL) and PMP-methanol (0.4 mol/L, 500 μL) were added to 250 μL of mixed standard and sample solutions. The reaction was carried out at 70 °C for 1 h. HCl (0.3 mol/L, 500 μL) and chloroform (1 mL) were sequentially added following the reaction, vortexed for 1 min, centrifuged at 3000 rpm for 10 min, and the supernatant was collected. This was repeated three times. The HPLC used the Xtimate C18 column (4.6 × 200 mm, 5 μm), column temperature at 30 °C, with a flow rate of 1.0 mL/min and an injection volume of 20 μL. The mobile phase was a mixture of 0.05 mol/L potassium dihydrogen phosphate solution (pH 6.70) and acetonitrile in a ratio of 83:17.

### 2.4. Molecular Weight Determination

The molecular weight of *L. bulgaricus* 1.0207 EPS and its homogeneity were determined by Gel Permeation Chromatography (GPC) [23]. A solution was prepared by dissolving 10 mg of *L. bulgaricus* 1.0207 EPS sample in 1 mL of deionized water, followed by filtration through a 0.45 μm membrane. The mobile phase was 0.1 M NaNO_3_ + NaN_3_ solution at a rate of 0.6 mL/min, column temperature at 35 °C. The instrument was equipped with a Shimadzu RID-20 oscillometric refractive detector and a TOSOH TSKgel GMPWXL aqueous gel chromatography column.

### 2.5. Infrared Spectral Analysis

The functional groups and glycosidic bonds in the *L. bulgaricus* 1.0207 EPS molecules were evaluated by Fourier transform infrared spectroscopy (FT-IR) (Shimadzu-8400s, Shimadzu, Japan). EPS and KBr (in a ratio of 1:100) were ground and compressed into transparent sheets for infrared spectral analysis, covering the spectral range from 400 cm^−1^ to 4000 cm^−1^.

### 2.6. Antioxidant Activity Assay of L. bulgaricus 1.0207 EPS In Vitro

#### 2.6.1. DPPH Radical Scavenging Activity

The DPPH radical scavenging activity was determined using the Zhao et al. method [24]. Specifically, 1 mL of different concentrations of EPS sample solution (0.5–4 mg/mL) was added to 2.0 mL of a 0.05 mM DPPH ethanol solution. The mixture was thoroughly combined and incubated for 30 min at room temperature, protected from light. The control group comprised deionized water and DPPH solution, while the blank group consisted of ethanol and sample solution. After centrifugation at 8000 rpm for 10 min, the absorbance of three replicate samples was measured at 517 nm. In the assays, vitamin C (Vc) was used as the positive control due to its known antioxidant properties [25], serving as a benchmark for evaluating the antioxidant capacity of the EPS samples.

$$Scavenging\;activity\;\%=1-\frac{A_{sample}-A_{blank}}{A_{control}}\times 100$$

Note: A_sample_: the absorbance of each sample; A_control_: the absorbance of the control group; A_blank_: the absorbance of the blank group.

#### 2.6.2. Hydroxyl Radical Scavenging Activity

The hydroxyl radical scavenging activity was measured as described by Min et al. [26] The reaction was initiated by adding 1 mL of 20 mmol/L H_2_O_2_ to a mixture comprising 1.0 mL of 0.1 M phosphate buffer saline (PBS) at pH 7.4, 1.0 mL of 2.5 mmol/L 1,10-phenanthroline, 1.0 mL of 2.5 mmol/L FeSO_4_, and 1.0 mL of EPS samples at varying concentrations (0.125–4.0 mg/mL). The mixture was then incubated at 37 °C for 90 min. Vc was used as a positive control, and the absorbance of the three replicate samples was measured at 536 nm.

$$Scavenging\;activity\;\%=\frac{A_{sample}-A_{blank}}{A_{control}-A_{blank}}\times 100$$

Note: A_sample_: the absorbance of each sample; A_blank_: the absorbance of the mixture without EPS sample; A_control_: the absorbance of the mixture without EPS sample and H_2_O_2_.

#### 2.6.3. Superoxide Anion Scavenging Activity

The superoxide anion scavenging activity was measured using the Wang et al. method [27]. First, 0.1 mL of EPS samples with different concentrations (0.125–4.0 mg/mL) and 2.8 mL of 0.05 mol/L Tris-HCl buffer (pH 8.2) were added to 100 μL of 0.05 mol/L o-toluene trisol. The mixture was incubated at 37 °C with shaking for 4 min under light-proof conditions. The reaction was then immediately terminated by adding 8 mol/L HCl. Deionized water was used as a control and Vc was used as a positive control. The absorbance of three replicate samples was measured at 325 nm.

$$Scavenging\;activity\;\%=\left(1-\frac{A_{sample}}{A_{blank}}\right)\times 100$$

Note: A_sample_: the absorbance of each sample; A_blank_: the absorbance of the mixture without EPS sample

#### 2.6.4. ABTS Radical Scavenging Capacity

The ABTS scavenging capacity of EPS solutions was determined using the ABTS kit (Beyotime, Shanghai, China), which employs the TEAC (Trolox Equivalent Antioxidant Capacity) method. The 10 μL EPS solution was added to the 200 μL ABTS radical cation solution, and after 5 min, the absorbance of the solution was measured at 734 nm. The ABTS radical scavenging capacity was calculated according to the kit instructions.

### 2.7. Effects of L. bulgaricus 1.0207 EPS against Hydrogen Peroxide-Induced Oxidative Damage in IPEC-J2 Cells

#### 2.7.1. CCK-8 Activity Assay

IPEC-J2 cells were cultured in 96-well plates. After the cells had adhered to the wall and reached a density of 1 × 10^5^/well, cells were treated according to the groups in Table 1. The effects of different H_2_O_2_ and EPS concentrations on the viability of IPEC-J2 cells, respectively, and the effects of different EPS concentrations on the viability of H_2_O_2_-treated IPEC-J2 cells were examined by the cell counting kit (CCK)-8 (Meilun Bio, Dalian, China) assay.

#### 2.7.2. IPEC-J2 Cellular Antioxidant Capacity Assay

IPEC-J2 cells were cultured in 6-well plates at a cell density of 1 × 10^6^/well. Cells were treated according to the groups in Table 2. The EPS groups were treated with different concentrations of EPS medium for 12 h, and the C and H_2_O_2_ groups were treated with normal cell medium for 12 h, finally, the cells were treated with 1000 μmol/L H_2_O_2_ for 4 h. Following the kit (Nanjing Jiancheng Bioengineering Institute, Nanjing, China) instructions, cell supernatants were collected for lactate dehydrogenase (LDH) and glutathione peroxidase (GSH-Px) assays. Additionally, adherent cells were collected for ultrasonic pulverization and determined the Superoxide dismutase (SOD), malondialdehyde (MDA), catalase (CAT), and total antioxidant capacity (T-AOC). Intracellular ROS were also measured by the kit (Nanjing Jiancheng Bioengineering Institute, Nanjing, China).

#### 2.7.3. Quantitative RT-PCR

The mRNA expression of antioxidant enzymes SOD, CAT, HO-1, GPX, and NQO1 was determined regarding the method of Guan et al. [28]. The mRNA expression of Nrf2, Keap1, and tight junction proteins (ZO-1, Occludin, Claudin1) were determined by RT-PCR as well. Total RNA was extracted from the cells and cDNA was synthesized according to the method provided by the kit. RT-PCR was determined using the QuantStudio 3 Real-Time PCR software version 1.4. The primer details can be found in Table S1. The relative expressions of genes were analyzed using the 2^−ΔΔCt^ calculation method and Gapdh was used as the internal reference gene.

#### 2.7.4. Western Blotting

The expression levels of related proteins in the Keap1/Nrf2 signaling pathway and key regulators of cell apoptosis were determined using western blotting. Total cellular proteins were extracted for western blotting analysis using the method of Guan [28]. Protein concentration was determined with a BCA kit (Meilun Bio, Dalian, China). Protein separation was performed by SDS-PAGE and transferred to PVDF membranes, blocked for 1–1.5 h. Membranes were incubated overnight with primary antibodies at 4 °C and washed with TBST three times. After that, diluted secondary antibody was added for incubation at room temperature for 30 min and then washed with TBST four times. Finally, the optical density of the target strip was analyzed by the Gel-Pro Analyzer version 4.0 software.

### 2.8. Statistical Analysis

This study ensured authenticity and reliability by conducting repetitive experiments with at least three independent groups. The results were expressed as mean ± standard deviation ($\bar{X}\pm SD$). The data were analyzed by one-way ANOVA (Analysis of Variance) and T-test using SPSS 24.0 software and Prism 9.3 software; *p* < 0.05 was considered significant. The graphs were plotted using Origin 8.0, Prism 9.3, and Excel 2021.

## 3. Results

### 3.1. Monosaccharide Composition and Molecular Weight of L. bulgaricus 1.0207 EPS

**Figure 1:**
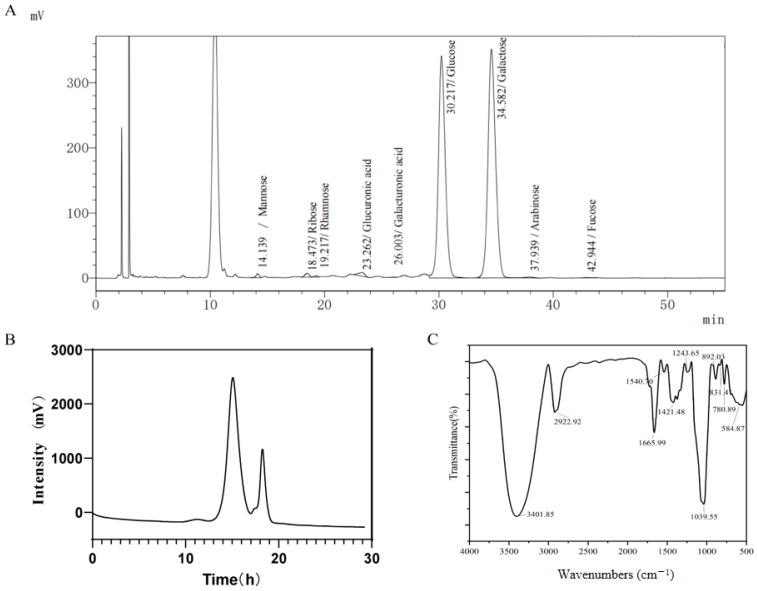
The structural composition of *L. bulgaricus* 1.0207 EPS. (**A**) Results of the determination of the monosaccharide composition of EPS by HPLC; (**B**) Results of molecular weight determination of EPS by GPC; (**C**) Evaluation of characteristic groups and glycosidic bonds in EPS molecules by FT-IR.

The EPS of *L. bulgaricus* 1.0207 comprised predominantly mannose, rhamnose, glucuronic acid, galacturonic acid, glucose, galactose, arabinose, and fucose (Figure 1A), with glucose and galactose collectively constituting 98.21% of the total polysaccharide content, exhibiting a molar ratio of 1.053:1. The weight-average molecular weight (Mw) of *L. bulgaricus* 1.0207 EPS was 4.06 × 10^4^ Da, while the number-average molecular weight (Mn) was 1.59 × 10^4^ Da. Additionally, the polydispersity coefficient (Mw/Mn) was calculated to be 2.57. The results from the GPC suggested inhomogeneity within *L. bulgaricus* 1.0207 EPS, as depicted in Figure 1B. This observation indicated the existence of distinct molecules within the *L. bulgaricus* 1.0207 EPS systems that have not undergone aggregation, with significant disparities in their molecular weights. Furthermore, many EPS with varying molecular weights within the system were corroborated by the large Mw/Mn of *L. bulgaricus* 1.0207 EPS.

### 3.2. Functional Group Analysis of L. bulgaricus 1.0207 EPS

The functional group composition of *L. bulgaricus* 1.0207 EPS is depicted in Figure 1C, revealing distinct absorption peaks characteristic of polysaccharides. A prominent absorption peak was detected at 3401.85 cm^−1^, arising from the stretching vibration of -OH groups, indicating the abundant presence of hydroxyl groups within the *L. bulgaricus* 1.0207 EPS sample [29]. The prominent absorption peak observed at 2922.92 cm^−1^ was due to the stretching vibration of C–H bonds. Meanwhile, minor absorption peaks detected in the range of 1200–1400 cm^−1^ could be attributed to variable-angle vibrations of C–H bonds as well. The absorption peak at 1665.99 cm^−1^ may signify the asymmetric stretching vibration of carbonyl groups, *L. bulgaricus* 1.0207 EPS may contain a minor fraction of uronic acid, aligning with the compositional findings of monosaccharides. The minor absorption peak observed at 1243 cm^−1^ was due to the stretching vibration of the -OH bonds within the carboxyl groups. The spectral peak at 1421.48 cm^−1^ falls within the range of 1398 cm^−1^ to 1443 cm^−1^, suggesting an association with the stretching vibration of the C-O bonds. Moreover, the strong absorption peak at 1039.55 cm^−1^, positioned in the spectral range of 950 cm^−1^ to 1200 cm^−1^, indicated the stretching vibrations associated with the C-O-C bonds in the pyran ring and the -OH bonds [30]. The absorption peak at 892.03 cm^−1^ suggested the presence of α-pyranose [31]. A series of absorption peaks around 584.87 cm^−1^ suggested the existence of α-glycosidic linkages within the *L. bulgaricus* 1.0207 EPS.

### 3.3. Antioxidant capacity of L. bulgaricus 1.0207 EPS In Vitro

**Figure 2:**
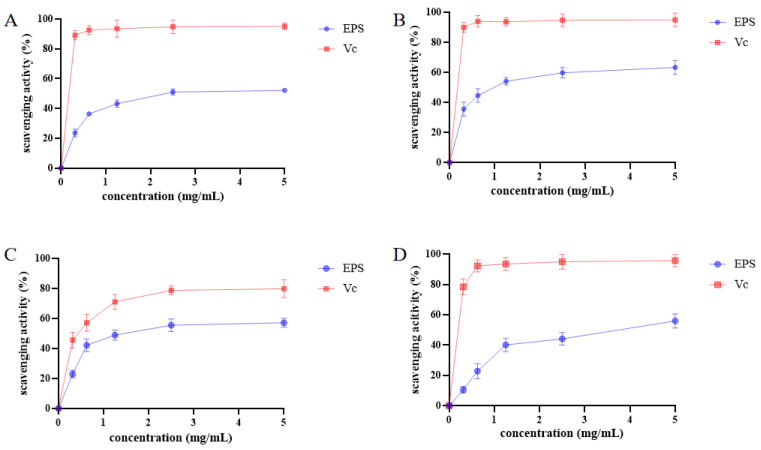
Antioxidant properties of *L. bulgaricus* 1.0207 EPS. (**A**) The EPS sample was mixed with DPPH ethanol solution, reacted for 30 min, centrifuged, and then the absorbance at 517 nm was measured to calculate DPPH scavenging activity; (**B**) The EPS sample was incubated with H_2_O_2_, PBS, 1,10-phenanthroline, and FeSO_4_ for 90 min, and hydroxyl radical scavenging was assessed by measuring absorbance at 536 nm; (**C**) The EPS sample was mixed with Tris-HCl buffer and o-toluidine, shaken for 4 min, then HCl was added to immediately terminate the reaction. Superoxide anion scavenging activity was assessed by measuring absorbance at 325 nm; (**D**) EPS was added to ABTS radical cation solution, and the absorbance at 734 nm was measured after 5 min to determine ABTS scavenging activity.

The antioxidant capacity of *L. bulgaricus* 1.0207 EPS in vitro is depicted in Figure 2. The DPPH radical scavenging ability of *L. bulgaricus* 1.0207 EPS exhibited a gradual increase with increasing EPS concentration, reaching a maximum scavenging rate of 56.8% at a concentration of 2.5 mg/mL, which was 58.9% of that observed for Vc at the same concentration. Similarly, the hydroxyl radical scavenging capacity was enhanced with increasing EPS concentration, reaching a maximum scavenging rate of 63.6% at a concentration of 5 mg/mL, which accounted for 66.7% of the same concentration of Vc. The superoxide anion scavenging capacity of *L. bulgaricus* 1.0207 EPS closely approached that of Vc, reaching 57.2%, which accounted for 72.6% of the capacity observed for Vc at the same concentration. The scavenging ability of *L. bulgaricus* 1.0207 EPS for ABTS radicals remained low within sample concentrations ranging from 0 to 1.25 mg/mL. However, the scavenging activity increased with the concentration of EPS, reaching 55.9% at a concentration of 5 mg/mL, which amounted to 58.3% of the scavenging activity of Vc at the equivalent concentration. Indeed, *L. bulgaricus* 1.0207 EPS demonstrated excellent antioxidant activity.

### 3.4. Effects of L. bulgaricus 1.0207 EPS on H_2_O_2_-Induced Cytotoxicity in IPEC-J2 Cells

**Figure 3:**
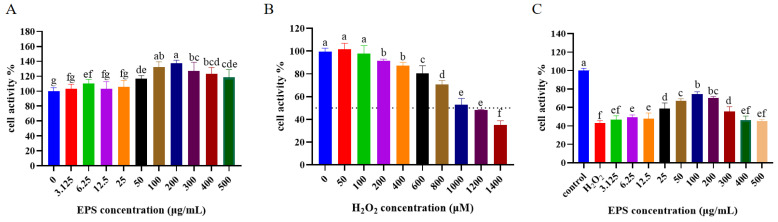
Effect of different EPS and H_2_O_2_ concentrations on the activity of IPEC-J2 cells as determined by CCK-8 dye. (**A**) effect of different concentrations of EPS on cell activity; (**B**) effect of different concentrations of H_2_O_2_ on cell activity; (**C**) effect of different EPS concentrations on cell activity recovery after oxidative stress caused by H_2_O_2_. Different lowercase letters indicate significant differences between groups (*p* < 0.05).

After treating the cells with *L. bulgaricus* 1.0207 EPS for 12 h, cell activity significantly improved with EPS concentrations ranging from 50 to 500 μg/mL compared to the blank group (EPS concentration of 0 μg/mL) (*p* < 0.05) (Figure 3A). The cell activity demonstrated a positive correlation with EPS concentration in the range of 50–200 μg/mL but decreased when the EPS concentration exceeded 200 μg/mL. When the cells were treated with H_2_O_2_, cell activity exhibited a significant decrease at H_2_O_2_ concentrations surpassing 600 μM (*p* < 0.05), and reaching a minimum cell activity of 38% at an H_2_O_2_ concentration of 1400 μM (Figure 3B). As shown in Figure 3B, 1000 µM H_2_O_2_ reduced cell viability to approximately 50% after 4 h, we selected 1000 µM H_2_O_2_ for establishing the oxidative damage model. Figure 3C illustrated the impact of varying EPS concentrations on the recovery of IPEC-J2 cell activity. Compared with the H_2_O_2_ group, EPS notably enhanced cell activity (*p* < 0.05), they were reaching the maximum proliferative activity of 69% at an EPS concentration of 100 μg/mL. Cell activity significantly decreased when EPS concentrations exceeded 200 μg/mL (*p* < 0.05).

### 3.5. Effect of L. bulgaricus 1.0207 EPS on Antioxidant Capacity in H_2_O_2_-Induced IPEC-J2 Cells

**Figure 4:**
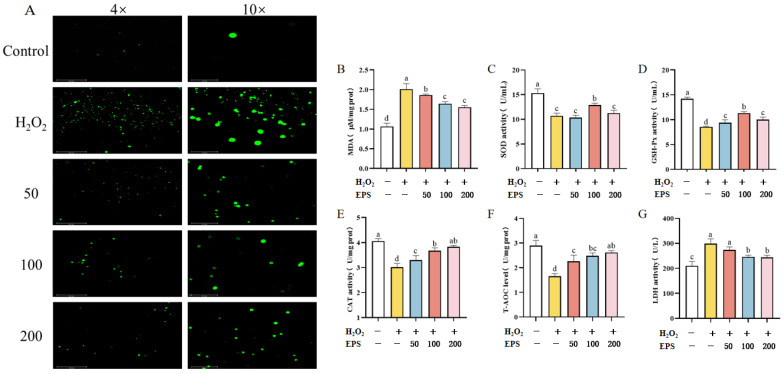
Effect of EPS on antioxidant indexes in oxidatively stressed IPEC-J2 cells. (**A**) DCF-DA staining to observe the changes of ROS levels in each group of cells at different multiplicities; ROS in cells showed green fluorescence under DCFH-DA staining (**B**) Collection of adherent cells for ultrasonic disruption followed by measurement of their MDA levels using kits; (**C**) Collection of adherent cells for ultrasonic disruption followed by measurement of their SOD levels using kits; (**D**) Detection of GSH-Px levels in cell supernatants using kits; (**E**) Collection of adherent cells for ultrasonic disruption followed by measurement of their CAT levels using kits; (**F**) Collection of adherent cells for ultrasonic disruption followed by measurement of their T-AOC levels using kits; (**G**) Detection of LDH levels in cell supernatants using kits. Different lowercase letters indicate significant differences between groups (*p* < 0.05).

The accumulation of H_2_O_2_ in the cell would affect the antioxidant capacity of the cell. ROS within the cells exhibited a green fluorescent signal under DCFH-DA staining conditions. A large range of green fluorescence was observed in the cells of the H_2_O_2_ group compared to the control group (Figure 4A). The area of green fluorescence notably decreased in the 50, 100, and 200 μg/mL EPS groups compared to the H_2_O_2_ group (*p* < 0.05). Figure 4B–G showed the effect of various treatments on the antioxidant enzyme activities in each group of cells. The activities of SOD, GSH-Px, CAT, and T-AOC were significantly reduced in the cells following H_2_O_2_ treatment when compared to the group without the addition of H_2_O_2_ and EPS (*p* < 0.05). Meanwhile, both cellular MDA and LDH levels in cell supernatants were significantly increased (*p* < 0.05). There were significantly improved cellular antioxidant enzyme activities in cells treated with EPS for 12 h when compared to the groups only with the addition of H_2_O_2_ (*p* < 0.05). Moreover, concentrations of 100 μg/mL and 200 μg/mL EPS demonstrated significantly greater improvements compared to 50 μg/mL (*p* < 0.05). There was no significant difference between the effects of 100 μg/mL and 200 μg/mL EPS (*p* > 0.05).

**Figure 5:**
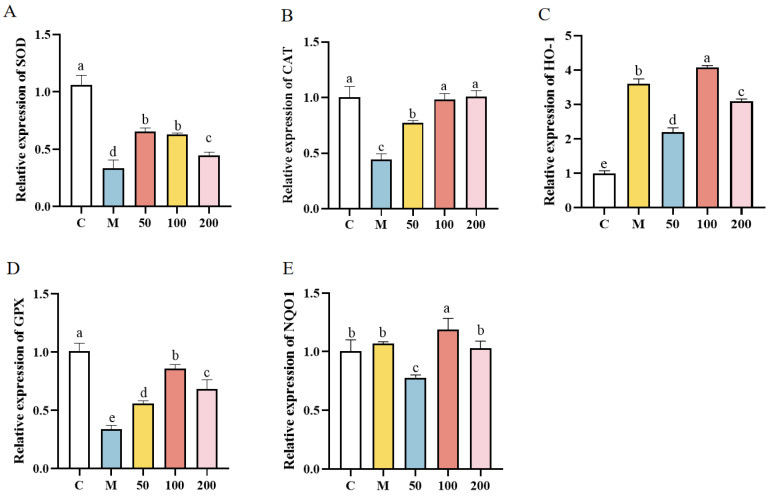
Effect of EPS on the expression of antioxidant genes in oxidatively stressed IPEC-J2 cells detected by qPCR. (**A**) Relative expression of SOD mRNA; (**B**) Relative expression of CAT mRNA; (**C**) Relative expression of HO-1 mRNA; (**D**) Relative expression of GPX mRNA; (**E**) Relative expression of NQO1 mRNA. Different lowercase letters indicate significant differences between groups (*p* < 0.05).

The expression of antioxidant genes in IPEC-J2 cells was depicted in Figure 5. In comparison to the control group, the mRNA expression of CAT, SOD, GPX, HO-1, and NQO1 was consistently observed to be significantly downregulated across all cell groups following treatment with H_2_O_2_ (*p* < 0.05). The expression of each gene was significantly elevated in the cells of the EPS group compared to those in the H_2_O_2_ group (*p* < 0.05). The mRNA expression of CAT in the 100 µg/mL and 200 µg/mL EPS groups was not significantly different from the control group (*p* > 0.05). The expression of HO-1 and NQO1 in the EPS groups was significantly higher than that in both the control and H_2_O_2_ groups (*p* < 0.05). The mRNA expression levels of HO-1 and NQO1 in the 100 µg/mL EPS group were the highest among all groups. This suggested a potentially greater activation of the Nrf2/HO-1 signaling pathway in the 100 µg/mL EPS group. *L. bulgaricus* 1.0207 EPS pretreatment effectively reduced the accumulation of intracellular ROS while enhancing the antioxidant capacity of the cells.

### 3.6. Effect of L. bulgaricus 1.0207 EPS on Keap1/Nrf2 Pathway in H_2_O_2_-Induced IPEC-J2 Cells

**Figure 6:**
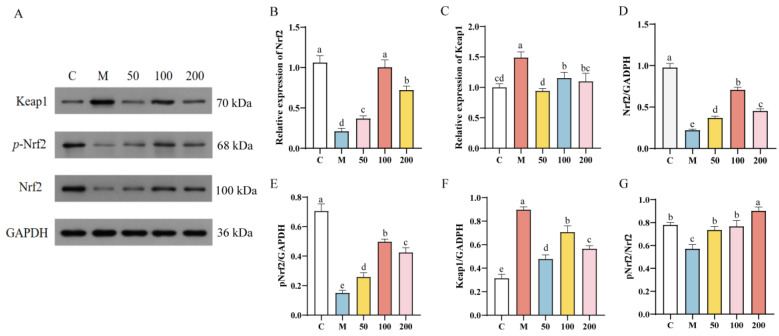
Effect of EPS on Keap1/Nrf2 pathway in oxidatively stressed IPEC-J2 cells. (**A**) Detection of protein expression related to the Keap1/Nrf2 signaling pathway using Western blotting; (**B**) Relative expression of Nrf2 mRNA; (**C**) Relative expression of Keap1 mRNA; (**D**) Relative protein expression of Nrf2; (**E**) Relative protein expression of pNrf2; (**F**) Relative protein expression of Keap1; (**G**) Relative protein expression of pNrf2/Nrf2. Different lowercase letters indicate significant differences between groups (*p* < 0.05).

The impact of *L. bulgaricus* 1.0207 EPS on the expression of genes and proteins related to the Keap1/Nrf2 signaling pathway in H_2_O_2_-induced IPEC-J2 cells is illustrated in Figure 6. The relative expression of Nrf2 mRNA was significantly downregulated (*p* < 0.05), while Keap1 mRNA was significantly upregulated (*p* < 0.05) in the group treated only with H_2_O_2_ when compared with the control group. However, compared with the H_2_O_2_ group, Nrf2 mRNA expression in the EPS group exhibited a significant increase (*p* < 0.05), while Keap1 mRNA expression was significantly decreased (*p* < 0.05) (Figure 6A,B). The results of RT-qPCR indicated that *L. bulgaricus* 1.0207 EPS activated the Keap1/Nrf2 signaling pathway, with the most pronounced activation observed in the 100 µg/mL EPS group. Likewise, Western blot results corroborated this finding, as depicted in Figure 6C–G. Compared to the control group, the protein expression levels of Nrf2, *p*Nrf2, and *p*Nrf2/Nrf2 in the H_2_O_2_ group exhibited a significant decrease (*p* < 0.05), whereas the protein expression of Keap1 showed a significant increase (*p* < 0.05). Conversely, the protein expression levels of Nrf2, *p*Nrf2, and *p*Nrf2/Nrf2 were significantly increased in the EPS group compared to the H_2_O_2_ group (*p* < 0.05), while the protein expression level of Keap1 was significantly decreased (*p* < 0.05). These findings indicate that *L. bulgaricus* 1.0207 EPS enhanced the expression of *p*-Nrf2 within the nucleus and improved the expression of antioxidant enzyme genes by activating the Keap1/Nrf2 signaling pathway.

### 3.7. Effect of L. bulgaricus 1.0207 EPS on Apoptotic Proteins in H_2_O_2_-Induced IPEC-J2 Cells

**Figure 7:**
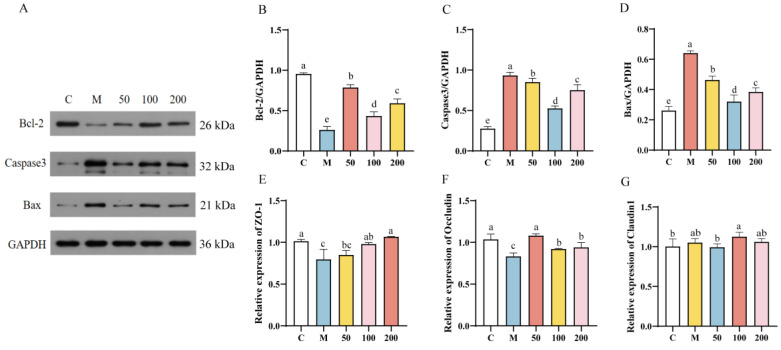
Effect of EPS on the expression of apoptotic proteins and genes of tight junction proteins in oxidatively stressed IPEC-J2 cells. (**A**) Detection of protein expression of apoptotic genes using Western blotting; (**B**) Relative protein expression of Bcl-2; (**C**) Relative protein expression of Caspase3; (**D**) Relative protein expression of Bax. (**E**) Relative protein expression of ZO-1; (**F**) Relative protein expression of Occludin; (**G**) Relative protein expression of Claudin1. Different lowercase letters indicate significant differences between groups (*p* < 0.05).

Oxidative stress is concomitant with apoptosis, the protein expression of apoptotic genes in IPEC-J2 cells was depicted in Figure 7A–D. The protein expression of Bcl-2 was significantly reduced in the H_2_O_2_ group compared to the control group (*p* < 0.05), while the protein expression of Caspase3 and Bax was significantly increased (*p* < 0.05). Compared to the H_2_O_2_ group, the EPS-pretreated groups exhibited a significant reversal in protein expression (*p* < 0.05), which was closer to that of the control group. The 100 µg/mL EPS group exhibited significantly lower expression levels of Caspase3 and Bax proteins compared to both the 50 µg/mL and 200 µg/mL EPS groups (*p* < 0.05). Additionally, the 50 µg/mL EPS group showed significantly higher expression of the Bcl-2 protein compared to both the 100 µg/mL and 200 µg/mL EPS groups (*p* < 0.05). Therefore, *L. bulgaricus* 1.0207 EPS significantly reduced the expression of apoptotic proteins and enhanced cell survival rates.

### 3.8. Effect of L. bulgaricus 1.0207 EPS on the Intestinal Barrier in H_2_O_2_-Induced IPEC-J2 Cells

H_2_O_2_ could cause intestinal barrier damage in IPEC-J2 cells, the mRNA expression of tight junction proteins (ZO-1, Occludin, Claudin1) in cells is illustrated in Figure 7E–G. The mRNA expression of ZO-1 and Occludin was notably reduced in the H_2_O_2_ group compared to the control group (*p* < 0.05). However, the EPS groups exhibited a significant restoration in ZO-1 and Occludin mRNA expression levels in comparison to the H_2_O_2_ group (*p* < 0.05). The mRNA expression levels of ZO-1 increased with EPS concentration, reaching a significant elevation in the 200 µg/mL EPS group compared to the 50 µg/mL EPS group (*p* < 0.05). There was no significant difference observed between the 200 µg/mL EPS group and the control group (*p* > 0.05). Additionally, the mRNA expression levels of Occludin in the EPS group exhibited a significant increase compared to the H_2_O_2_ group (*p* < 0.05). No significant difference was observed between the 50 µg/mL EPS group and the control group (*p* > 0.05). There was no significant difference in Claudin1 mRNA expression between the groups (*p* > 0.05). These findings suggest that *L. bulgaricus* 1.0207 EPS has the potential to mitigate intestinal barrier damage by regulating the mRNA expression levels of ZO-1 and Occludin.

## 4. Discussion

Oxidative stress is a state in which intracellular redox reactions are disturbed, resulting in the accumulation of ROS in the cell [32]. Oxidative stress causes oxidative damage to proteins and lipids, which could lead to cellular senescence, apoptosis, or disease [33]. Treatment with appropriate antioxidants can improve human health by alleviating oxidative stress damage. The potential antioxidant activity of EPS in probiotics has been reported in recent years [34,35,36]. This study analyzed the synthesis pathway and the fundamental structure of *L. bulgaricus* 1.0207 EPS and explored the mechanism of oxidative stress mitigation by *L. bulgaricus* 1.0207 EPS in vitro.

The structure of *L. bulgaricus* 1.0207 EPS was analyzed in this study, focusing on monosaccharide composition, molecular weight, and functional groups. The monosaccharides of *L. bulgaricus* 1.0207 EPS are mainly glucose and galactose, with small amounts of mannose, glucuronic acid, and other monosaccharides. The findings were consistent with the monosaccharide composition results reported for *L. bulgaricus* SRFM-1 by Tang [6], where both glucose and galactose were identified as the predominant components. Sandrine Petry et al. [37] demonstrated that the monosaccharide composition of *L. bulgaricus* 1187 EPS was galactose and glucose. The molecular weight of *L. bulgaricus* 1.0207 EPS was determined to be 4.06 × 10^4^ Da. Most studies have indicated that the molecular weight of Lactic acid bacterial EPS typically falls within the range of 1 × 10^4^ to 1 × 10^6^ Da [38]. The molecular weight of *L. bulgaricus* 1.0207 EPS was in this range, indicating that it is a low molecular weight polysaccharide. Low molecular weight polysaccharides have been shown to exhibit various immunological and antioxidant activities. Sheng et al. [39] examined the antioxidant activity of polysaccharides with different molecular weights, such as DPPH scavenging capacity and hydroxyl radical scavenging capacity, revealed that low molecular weight polysaccharides exhibited enhanced antioxidant properties. Surayot et al. [40] discovered that after partial acid hydrolysis of *Weissella* TISTR1498 EPS, which changed its molecular weight to 7 × 10^4^ Da, it significantly increased the production of nitric oxide (NO) and various immune factors in RAW264.7 cells. In this study, we found that *L. bulgaricus* 1.0207 EPS demonstrated a strong antioxidant capacity. It effectively neutralized free radicals and reduced cellular damage induced by oxidative stress. Tang et al. [6] found that the polysaccharide extracted from *L. bulgaricus* SRFM-1 exhibited robust scavenging activity against superoxide radicals, hydroxyl radicals, DPPH radicals, and ferrous ions. These findings are in line with our results.

H_2_O_2_ can lead to adaptive stress responses in organisms by modulating gene expression within the Keap1/Nrf2 and NF-κB signaling pathways under appropriate concentration conditions [41]. However, it can lead to oxidative stress when the concentration of H_2_O_2_ exceeds a certain threshold [41]. Therefore, H_2_O_2_ was chosen as the oxidant in this study to induce direct oxidative damage to the cells. The excessive production of ROS disrupts the redox balance in the organism, which may disrupt endogenous antioxidant defense mechanisms and result in intracellular damage [42]. Antioxidant enzymes such as SOD, GSH, and CAT play crucial roles in promoting the degradation of ROS. Elevated levels of MDA, a product of lipid peroxidation, indicate the degree of oxidative damage to the cell. Our experimental results demonstrated that compared to the control group, the cells in the H_2_O_2_ group exhibited a pronounced aggregation of ROS green fluorescence, a decrease in mRNA expression levels of the antioxidant enzymes SOD, CAT, and GSH-Px. Additionally, there was a significant elevation in the levels of MDA and LDH in the H_2_O_2_ cells (*p* < 0.05), suggesting severe oxidative damage and a decline in antioxidant capacity. However, the EPS group reversed these changes, exhibiting a reduction in ROS green fluorescence, an increase in both antioxidant enzyme activity and mRNA expression, a significant decrease in LDH and MDA levels, and a notable enhancement in antioxidant capacity compared to the model group (*p* < 0.05).

The Keap1/Nrf2 signaling pathway is one of the mechanisms in cellular responses to oxidative stress. The lack of activation in the Keap1/Nrf2 signaling pathway results in impaired cellular redox homeostasis, exacerbating oxidative damage and culminating in cell death [43]. This study revealed that the Keap1/Nrf2 signaling pathway was activated in the cells treated with *L. bulgaricus* 1.0207 EPS, with an increase in the protein expression levels of Nrf2, and *p*Nrf2 and a decrease in Keap1.

Oxidative stress can trigger apoptosis, a form of programmed cell death. Oxidative stress disrupts the balance of Bcl-2 family proteins, leading to increased mitochondrial membrane permeability and the release of cytochrome c, which ultimately activates caspase-3 and initiates apoptosis [44]. In our study, we observed a decrease in Bcl-2 protein expression, accompanied by elevated levels of caspase-3 and Bax proteins in the model group, in which apoptosis was facilitated. *L. bulgaricus* 1.0207 EPS effectively mitigated oxidative stress-induced apoptosis in IPEC-J2 cells, with a concentration of 100 µg/mL exhibiting the highest efficacy. Moreover, H_2_O_2_ can damage the intestinal barrier function by downregulating the mRNA expression of tight junction proteins, inducing apoptosis in intestinal epithelial cells [13]. Our experimental findings indicated a notable decrease in mRNA expression levels of Occludin and ZO-1, implying that oxidative stress impairs gut barrier integrity. The EPS group significantly enhanced the mRNA expression of tight junction protein, indicating that *L. bulgaricus* 1.0207 EPS can improve gut barrier stability.

The findings of this study indicated that *L. bulgaricus* 1.0207 EPS exhibited the potential to alleviate oxidative stress and promote cell survival rates. Li et al. [13] reported that *L. rhamnosus* GG EPS demonstrated notable antioxidant and antiapoptotic effects on IPEC-J2 cells, it effectively upregulated the expression of tight junction proteins, maintaining intestinal barrier stability, which was in agreement with us. This result is also corroborated by the report on the amelioration of oxidative stress by plant-derived compounds by Wang et al. [45]. Their study revealed that pretreatment with plant-derived compounds increased the antioxidant capacity of cells while activating Keap1/Nrf2 signaling pathway reduced apoptosis and resisted cellular oxidative stress damage.

## 5. Conclusions

In conclusion, *L. bulgaricus* 1.0207 EPS mitigated H_2_O_2_-induced oxidative stress through the Keap1/Nrf2 pathway and enhanced the gut barrier. *L. bulgaricus* 1.0207 EPS enhanced cellular antioxidant enzyme activities of SOD, GSH-Px, CAT, and T-AOC, improved the expression of antioxidant enzyme genes CAT, SOD, HO-1, and NQO1, and reduced intracellular ROS accumulation. Meanwhile, *L. bulgaricus* 1.0207 EPS reduced the expression of apoptotic proteins Caspase3, and Bax, and enhanced cell survival rates. These findings underscore the potential of *L. bulgaricus* 1.0207 EPS in treating oxidative stress-related intestinal diseases.

## Figures and Tables

**Table 1:** Cell viability assays for different treatment groups.

Study Content	Treat with H_2_O_2_	Treat with EPS
Effect of different H_2_O_2_ concentrations on the viability of IPEC-J2 cells	IPEC-J2 cells viability was determined after 4 h of treatment with 0, 50, 100, 200, 400, 600, 800, 1000, 1200, and 1400 μmol/L H_2_O_2_	
Effect of different EPS concentrations on the viability of IPEC-J2 cells		Cell viability was determined after 12 h of treatment with 0, 6.25, 12.5, 25, 50, 100, 200, 400, 600, and 800 μg/mL EPS
Effect of different EPS concentrations on the viability of IPEC-J2 cells treated with H_2_O_2_	IPEC-J2 cells were treated with 0, 6.25, 12.5, 25, 50, 100, 200, 400, 600, and 800 μg/mL EPS for 12 h and then added 1000 μmol/L H_2_O_2_ for 4h to determine the viability	

**Table 2:** Cell groupings.

Grouping	Treatment to Cells	
C	Normal cell medium 12 h	Normal cell medium 4 h
H_2_O_2_	Normal cell medium 12 h	Treat with 1000 μmol/L H_2_O_2_ 4 h
50	Cell medium with 50 μg/mL EPS 12 h	Treat with 1000 μmol/L H_2_O_2_ 4 h
100	Cell medium with 100 μg/mL EPS 12 h	Treat with 1000 μmol/L H_2_O_2_ 4 h
200	Cell medium with 200 μg/mL EPS 12 h	Treat with 1000 μmol/L H_2_O_2_ 4 h

## Data Availability

The data analyzed during the current study are available from the corresponding author upon reasonable request.

## Supplementary Materials

The following supporting information can be downloaded at: https://www.mdpi.com/article/10.3390/antiox13091150/s1.
