# Supplementary material for: Lactobacillus delbrueckii subsp. bulgaricus 1.0207 Exopolysaccharides Attenuate Hydrogen Peroxide-Induced Oxidative Stress Damage in IPEC-J2 Cells through the Keap1/Nrf2 Pathway
Source: Antioxidants (Basel). 2024 Sep 23;13(9):1150. doi: 10.3390/antiox13091150 (PMC11429245; doi:10.3390/antiox13091150)
Supplement: Supplementary file 1 [file antioxidants-13-01150-s001.zip › antioxidants-3180393-supplementary.pdf]

Supplementary Materials

Table S1. Primer Sequences of RT-qPCR.

Gene	Forward (5′- 3′)	Reverse (5′- 3′)	Accession No.
Keap1	AAACCGCCTCAACTCAGCAG	CTGGTCCTGACCATCGTAGC	XM_005654811.3
Nrf2	GGAGCTGTTGATCTGTTGCG	TCCATGTCCCTTGACAGCAA	XM_021075133.1
HO-1	TACCGCTCCCGAATGAACAC	GTCACGGGAGTGGAGTCTTG	NM_001004027.1
NQO1	GATCATACTGGCCCACTCCG	GAGCAGTCTCGGCAGGATAC	NM_001159613.1
SOD	GAGACCTGGGCAATGTGACT	CTGCCCAAGTCATCTGGTTT	NM_001190422.1
CAT	CAGATGAAGCATTGGAAGGAGC	TTGTCTCCTATCGGATTCCCAG	NM_214301.2
GPX	CCTAGCAGTGCCTAGAGTGC	CGCCCATCTCAGGGGATTTT	NM_214201.1
ZO-1	TTGATAGTGGCGTTGACA	CCTCATCTTCATCATCTTCTAC	XM_021098896.1
Occludin	CAGGTGCACCCTCCAGATTG	ATGTCGTTGCTGGGTGCATA	NM_001163647.2
Claudin-1	CTAGTGATGAGGCAGATGAA	AGATAGGTCCGAAGCAGAT	XM_005670262.3
Gapdh	GACAGCCGCATCTTCTTGTG	AATCCGTTCACACCGACCTT	XM_063285517.1
